# An fNIRS dataset for driving risk cognition of passengers in highly automated driving scenarios

**DOI:** 10.1038/s41597-024-03353-6

**Published:** 2024-05-28

**Authors:** Xiaofei Zhang, Qiaoya Wang, Jun Li, Xiaorong Gao, Bowen Li, Bingbing Nie, Jianqiang Wang, Ziyuan Zhou, Yingkai Yang, Hong Wang

**Affiliations:** 1 School of Vehicle and Mobility, Tsinghua University, Beijing, 100084 China; 2 School of Medicine, Tsinghua University, Beijing, 100084 China; 3 Department of Electrical and Electronic Engineering, Imperial College London, London, SW7 2AZ UK

**Keywords:** Mechanical engineering, Human behaviour

## Abstract

For highly autonomous vehicles, human does not need to operate continuously vehicles. The brain-computer interface system in autonomous vehicles will highly depend on the brain states of passengers rather than those of human drivers. It is a meaningful and vital choice to translate the mental activities of human beings, essentially playing the role of advanced sensors, into safe driving. Quantifying the driving risk cognition of passengers is a basic step toward this end. This study reports the creation of an fNIRS dataset focusing on the prefrontal cortex activity in fourteen types of highly automated driving scenarios. This dataset considers age, sex and driving experience factors and contains the data collected from an 8-channel fNIRS device and the data of driving scenarios. The dataset provides data support for distinguishing the driving risk in highly automated driving scenarios via brain-computer interface systems, and it also provides the possibility of preventing potential hazards in some scenarios, in which risk remains at a high value for an extended period, before hazard occurs.

## Background and Summary

The NHTSA’s National Center for Statistics and Analysis published a national motor vehicle crash causation survey, which was conducted from 2005 to 2007, and its results indicate that the critical reason for crashes is assigned to the driver in 94 percent (±2.2%) of cases^1^. Driving is not as safe or as easy as it should be, while distracted driving is on the rise. Waymo believe their technology could save thousands of lives now lost to traffic crashes every year^2^. However, some accidents have attracted researchers’ attention regarding the safety of highly autonomous vehicles. For example, a highly autonomous vehicle hit an overturned white truck due to misidentifying the white as cloud. Similarly, an incident has occurred in which a highly autonomous vehicle hit a pedestrian as it did not take consider the possibility of illegal road crossing by pedestrians^3^. In ISO/DIS 21448, the absence of unreasonable risk due to such potentially hazardous behaviours related to these functional insufficiencies is defined as the safety of the intended functionality (ISO21448)^4^. Humans are essentially a special kind of sensor and can perceive risks. It is therefore meaningful to study brain-computer interface systems based on human states to overcome functional deficiencies of automated driving algorithms.

Functional near-infrared spectroscopy (fNIRS) technology can provide data on oxy-hemoglobin change Δ*Hbo* and deoxy-hemoglobin change Δ*HbR*, which can indicate prefrontal activity. With the development of highly autonomous vehicles, an increasing number of researchers working on this topic are giving consideration to fNIRS-based driving^5–10^. Some researchers have conducted studies on prefrontal activity of human drivers based on fNIRS. The differences of mental workloads, which were measured based on fNIRS, between city-side environments and country-side environments were analyzed^11^, and it pointed out that higher bilateral middle frontal gyrus activation is present in demanding city environments than in less demanding country environments. Foy *et al*. examined the mental workload of human drivers caused by changes in the road environment, and it was shown that the increase in subjective ratings of mental workload caused by changes in road type were accompanied by increases in skin conductance, acceleration signatures and horizontal spread of search, such changes were accompanied by an increase in the concentration of oxygenated hemoglobin in the prefrontal cortex^12^. Besides, the prefrontal cortex activations caused by manipulations of mental workload and inhibitory control were explored^13^, and it shows that prefrontal cortex activity is associated with the mental workload required for overtaking cars. Huve *et al*.^14^ designed a brain—computer interface system that could analyse the brain activity of a user in real time and deduce the current driving mode of the car, although its classification accuracy was 61.7%, it showed the potential of developing brain—computer interface systems based on fNIRS in driving scenarios. As stated above, activity in the prefrontal cortex is related to mental workload, thus prefrontal cortex is the region of interest for our study. However, previous studies all focused on human drivers. Data is important for mechanism analysis. There are several well-known datasets, such as NASS-CDS^15^ and GIDAS^16^, which provide detailed information on human driver injuries and are used for studying and evaluating injury patterns and severity. Furthermore, there are also other datasets that focus on the psychological, physiological, and behavioral aspects of human drivers during driving tasks, such as a multi-modal human emotion dataset for driving tasks^17^ and a multimodal dataset for various forms of distracted driving^18^. The latter records the heart rates, breathing rates and eye tracking data of human drivers under four different conditions. Despite the existence of numerous well-known datasets for highly autonomous vehicles, no datasets exist with a focus on the mental activities of passengers in highly automated driving scenarios. fNIRS traditionally needs taking several seconds to provide insight into neural activation, and it often is used as a post-event analytical tool, beside some studies^19–21^ have explored fast optical response and initial dip as potential data methods for faster fNIRS data inferences. It has been concluded that prefrontal cortical hemoglobin oxygenation levels significantly increase, following self-reported perceived risk and traffic complexity, particularly during the hazardous scenario^22^, and we assume that objective driving risk of environment will cause changes in the prefrontal cortex activity of passengers. There are some scenarios, in which risk may remain at high value for an extended period, before a hazard occurs, such as one vehicle is running unsafely in an adjacent lane, causing a passenger to think, “it may collide with my car, and I should stay away from this vehicle”. Our objective is to provide data support for finding the differences in prefrontal cortex activity between low-risk and high-risk episodes by quantifying the risk of driving scenarios, and high-risk imply hazardous event will may occur. This research may provide a solution to prevent potential hazards and improve SOTIF based on brain-computer interface technology and fNIRS, in the future.

To compensate for the lack of data on passengers’ mental activities in highly automated driving scenarios, promote the research of passenger in-loop decision-making and improve SOTIF, we built an fNIRS dataset focusing on passengers’ mental activities in highly automated driving scenarios, and this dataset can be used to analyze the driving risk cognition of passengers. This dataset contains data from 20 participants collected in fourteen types of highly automated driving scenarios. This dataset contains the raw intensity data which can be converted into Δ*Hbo* and Δ*HbR* based on the modified Lambert-Beer law, and split points. Those split points are defined according to a risk field, this risk field may be calculated based on driving scenario data, and the driving scenario data refers to vehicle-related data, which are composed of the positions, velocities and accelerations of the ego vehicle and target vehicle (or pedestrian). Besides, the data of each scenario were divided into low-risk and high-risk episodes in accordance with one split point, and the rationality of the episode division was verified by a data summary chart. We have adjusted our data to be BIDs-complaint, and maded the data available at OpenNeuro under the 10.18112/openneuro.ds004973.v1.0.1. This dataset covers fourteen types of highly automated driving scenarios, and the data at four types of scenarios have been analyzed. Based on the data in this dataset for the scenario in which an surrounding vehicle cuts in from the left lane over a short distance scenario and an actual vehicle experiment of cut-in scenario, it was concluded that high risk may result in the passengers’ mental activity on prefrontal cortex change, and this study has been accepted for publication by the journal of Automotive Innovation as the manuscript “Shedding light on the prefrontal correlates of passengers’ mental activity based on fNIRS for high-level automated vehicles”; Besides, another study has found that the mental activities of passengers in Brodmann area 10 caused by driving scenario risk are very active, and there is a positive correlation between the data from four scenarios and another actual vehicle experiment^23^. Although, the participants are the same across the above publications, the data for other ten types of scenarios of in this dataset have never been analyzed. In this study, we would like to widely introduce this dataset and and make it available for open use, so that more people can perform more meaningful investigations.

## Methods

### Ethics statement

This study complies with the Declaration of Helsinki and later amendments of it. The content and procedures of this study were approved by the Institutional Review Board of Tsinghua University (Approval number: 20210102).

The data were collected from a driving simulator using a human factor signal acquisition system. Twenty participants completed this experiment, and all of them were free from any disease or predisposition for simulator sickness. Participants were voluntary after the task and informed consent have been explained, and they were further informed that they could terminate this experiment at any time without any type of penalty. The informed consent form included a notice that the results and data associated with this work might be published in academic journals or books. In the dataset, the real names of participants have been replaced characters and Arabic numerals, and the data contained personally identifiable information have been anonymized.

### Experiment

**Fig. 1:**
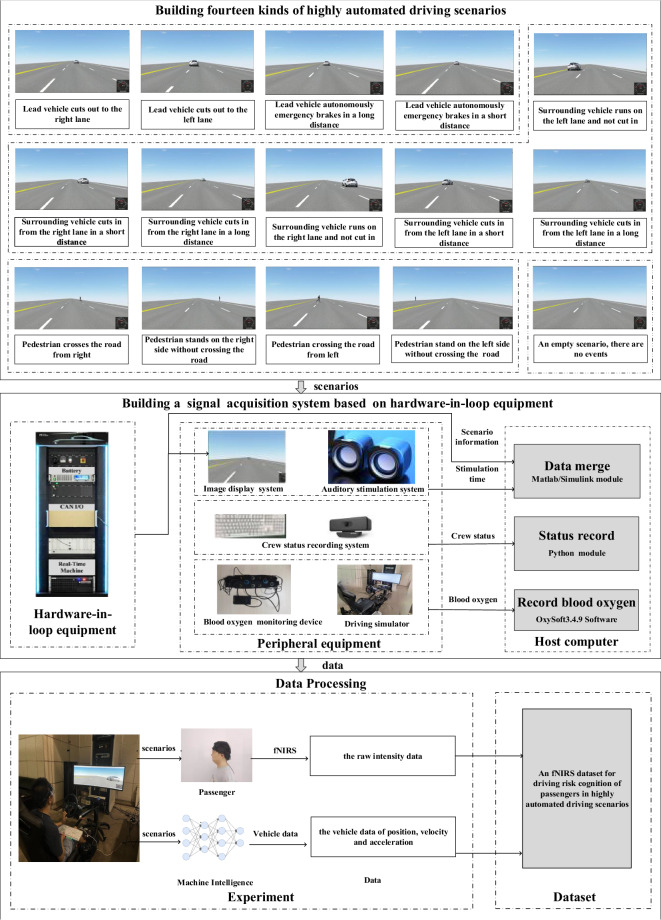
The process of building this dataset.

Each participant need to finish 12 tasks. For each task, participants need to sit in a driving simulator and focus on watching a different virtual test drive (VTD) segment, and they not free to perform other tasks, as this could disrupt their situation awareness. The participants act as a passenger and do not directly operate driving simulator. Participants is asked to rest for approximately 5 minutes at the end of each task, and during any one day, participants only take part in four tasks. Upon sensing danger or hearing a stimulating sound, he/she is required to press the keyboard. A few random stimulating sounds are added in each task, with the motivation of judging whether or not the participants are focusing on those task by comparing the time delays at which participants pressed the keyboard when they heard a stimulating sound. Each segment is composed of twenty-five scenarios that are randomly chosen from among fourteen types of highly automated driving scenarios. The process is shown in Fig. 1. It contains three parts, building fourteen types of highly automated driving scenarios, building a signal acquisition system and data processing.

#### Building fourteen types of highly automated driving scenarios

In this study, firstly, fourteen types of highly automated driving scenarios are built based on VTD software. VTD software is a commercial software and is owned by Hexagon, which is a Swedish company, and in our experiments, the version 2021 of this software is uesd. This software may be purchased at the following this link https://hexagon.com/company/contact-us. These scenarios consist of an ego vehicle, a target vehicle or pedestrian. Ego vehicle is one highly autonomous vehicle, and it is controlled by an algorithm in the VTD software. Lead vehicle or surrounding vehicle may cut in or brake urgently, and pedestrian may cross road. These fourteen types of scenarios belong to one of these three typical single-car scenarios: cut-in, emergency braking, and pedestrian crossing road. These fourteen types of highly automated driving scenarios are lead vehicle cuts out to the right lane, lead vehicle cuts out to the left lane, lead vehicle autonomously emergency brakes over a long distance, surrounding vehicle cuts in from the left lane over a short distance, surrounding vehicle cuts in from the right lane over a short distance, surrounding vehicle cuts in from the right lane over a long distance, lead vehicle autonomously emergency brakes over a short distance, surrounding vehicle cuts in from the left lane over a long distance, surrounding vehicle runs on the right land and does not cut-in, pedestrian crosses the road from right, pedestrian crosses the road from left, pedestrian stands on the left side without crossing the road, pedestrian stands on the right side without crossing the road, surrounding vehicle runs on left lane and does not cut-in. Secondly, twenty-five scenarios are randomly chosen from these fourteen types of highly automated driving scenarios to compose a VTD segment. Within the initial 1000 meters(ms) of each VTD segment there are no event thus allowing the participant to acclimatize to the simulated driving environment, ego vehicle simply moves straight ahead on a three-lane road at 70 km/h. When ego vehicle arrives at a trigger position, a target vehicle or pedestrian will take an action, such as cut-in, cut-out or cross road and so on. Finally, these VTD segments are deployed in a human factor signal acquisition system which will be introduced later. A total of twelve VTD segments are prepared, each segment is approximately 13 minutes. The data of the last scenario in each VTD segment are rejected, and only the data of the ahead twenty-four scenarios are extracted for data analysis. The order of the types of twenty-four scenarios in the twelve VTD segments are shown in Table 1, Seg *i* stands for the *i* th VTD segment, and S *i* stands for the *i* th scenario of fourteen types of highly automated driving scenarios, and No stands for the scenario order in a VTD segment. Detailed information about the fourteen types of highly automated driving scenarios is provided in Table 2, where the “Number” column represents the total number of times the corresponding type of driving scenario appears in the 12 tasks, that need to be completed by each participant.

**Table 1:** The order of scenario type in twelve VTD segments.

N0	Seg 1	Seg 2	Seg 3	Seg 4	Seg 5	Seg 6	Seg 7	Seg 8	Seg 9	Seg 10	Seg 11	Seg12
01	S11	S6	S10	S7	S10	S9	S10	S6	S11	S11	S10	S12
02	S13	S2	S6	S3	S8	S10	S8	S3	S7	S6	S3	S1
03	S1	S10	S5	S11	S1	S14	S9	S8	S4	S4	S8	S10
04	S3	S2	S13	S13	S10	S5	S3	S5	S13	S11	S7	S4
05	S6	S9	S10	S8	S6	S11	S6	S10	S4	S3	S10	S3
06	S2	S11	S7	S10	S4	S4	S11	S12	S14	S4	S2	S2
07	S9	S6	S8	S13	S3	S6	S9	S7	S10	S8	S6	S13
08	S10	S4	S11	S5	S6	S10	S9	S12	S8	S13	S5	S11
09	S10	S5	S10	S13	S11	S7	S11	S8	S10	S1	S11	S14
10	S5	S11	S14	S2	S6	S12	S12	S12	S10	S14	S10	S13
11	S14	S4	S10	S12	S3	S2	S14	S11	S9	S7	S5	S10
12	S13	S7	S12	S4	S5	S12	S13	S5	S12	S5	S6	S12
13	S8	S10	S7	S5	S7	S2	S4	S13	S6	S12	S4	S3
14	S6	S11	S4	S4	S10	S14	S13	S1	S11	S4	S14	S1
15	S12	S8	S9	S11	S13	S14	S6	S9	S4	S6	S5	S13
16	S14	S14	S10	S8	S6	S12	S11	S13	S5	S10	S6	S12
17	S4	S2	S4	S5	S10	S4	S10	S1	S11	S7	S10	S4
18	S5	S3	S10	S5	S4	S4	S13	S4	S9	S12	S5	S2
19	S12	S8	S14	S12	S10	S11	S2	S4	S11	S9	S6	S11
20	S4	S8	S7	S9	S5	S4	S5	S10	S8	S8	S8	S9
21	S11	S4	S1	S6	S9	S11	S13	S13	S1	S14	S5	S13
22	S2	S12	S11	S9	S5	S14	S3	S9	S5	S11	S14	S8
23	S13	S9	S3	S10	S14	S5	S2	S4	S2	S9	S13	S11
24	S5	S14	S11	S13	S7	S13	S7	S11	S8	S11	S9	S13

**Table 2:** The information of fourteen types of highly automated driving scenarios.

No	Shorthand of scenario name	Scenario name	Scenario Description	Number
1	Scenario 1	lead vehicle cuts out to the right lane	Lead vehicle runs at 50 km/h, when the distance between lead vehicle and ego vehicle is 50 m, lead vehicle cuts out to the right lane.	9
2	Scenario 2	lead vehicle cuts out to the left lane	Lead vehicle runs at 40 km/h, when the distance between lead vehicle and and ego vehicle is 25 m, lead vehicle cuts out to the left lane.	14
3	Scenario 3	lead vehicle autonomously emergency brakes over a long distance	Lead vehicle runs at 50 km/h, when the distance between lead vehicle and ego vehicle is 35 m, lead vehicle brake to 20 km/h. when the distance between lead vehicle and ego vehicle is 19 m, lead vehicle cuts out.	13
4	Scenario 4	surrounding vehicle cuts in from the left lane over a short distance	Surrounding vehicle runs at 40 km/h, when the distance between surrounding vehicle and ego vehicle is 25 m, surrounding vehicle cut in from the left lane.	28
5	Scenario 5	surrounding vehicle cuts in from the right lane over a short distance	Surrounding vehicle runs at 40 km/h, when the distance between surrounding vehicle and ego vehicle is 35 m, surrounding vehicle cut in from the right lane.	25
6	Scenario 6	surrounding vehicle cuts in from the right lane over a long distance	surrounding vehicle runs at 40 km/h, when the distance between surrounding vehicle and ego vehicle is 60 m, surrounding vehicle cut in from the right lane.	22
7	Scenario 7	lead vehicle autonomously emergency brakes over a short distance	Lead vehicle runs at 55 km/h, when the distance between lead vehicle and ego vehicle is 40 m, lead vehicle brake to 10 km/h. when the distance between lead vehicle and ego vehicle is 18 m, the lead vehicle cuts out.	14
8	Scenario 8	surrounding vehicle cuts in from the left lane over a long distance	Surrounding vehicle runs at 40 km/h, when the distance between surrounding vehicle and ego vehicle is 60 m, surrounding vehicle cut in from the left lane.	19
9	Scenario 9	surrounding vehicles runs on the right land and do not cut in	Surrounding vehicle runs at 40 km/h on the right land and does not cut in.	19
10	Scenario 10	pedestrian crosses the road from right	When the distance between ego vehicle and pedestrian is 55 m, pedestrian crosses crosses the road from right at 10.91 km/h	33
11	Scenario 11	pedestrian crosses the road from left	When the distance between ego vehicle and pedestrian is 120 m, pedestrian crosses crosses the road from left at 10.91 km/h.	31
12	Scenario 12	pedestrian stands on the left side without crossing the road	Pedestrian stand on the left side and does not cross the road	19
13	Scenario 13	pedestrian stands on the right side without crossing the road	Pedestrian stands on the right side and does not cross the road	25
14	Scenario 14	surrounding vehicle runs on left land and does not cut in	Surrounding vehicle runs at 40 km/h on left lane and does not cut in.	17

#### Building a human factor signal acquisition system

This human factor signal acquisition system is composed of a hardware-in-loop equipment, peripheral equipment and a host computer, as shown in Fig. 1. The hardware-in-loop equipment is a real-time system (Redhawk system), and it runs the VTD software. VTD software sends driving scenario data and exports VTD segments to a monitor for video visualization. The driving scenario data are compose of the positions, velocits and accelerations of ego vehicle and target vehicle (or pedestrian). The peripheral equipment includes one camera and one keyboard. The camera is a 4 K Ultra HD USB camera, and it is manufactured by HKVISION with a pixel resolution of 3840(horizontal) x 2160(vertucal). The host computer runs Matlab/Simulink module, Python module and OxySoft software. OxySofts software records the raw data of Δ*Hbo* and Δ*HbR*, which are measured by a blood oxygen monitoring device, besides, the OxySofts software also exports raw intensity data, those raw intensity data may be preprocessed based on specific needs to obtain preprocessed data of Δ*Hbo* and Δ*HbR* by Homer3^24^ or other softwares; The Matlab/Simulink module undertakes the communication between hardware-in-loop equipment and host computer, and the communication between host computer and OxySoft software. The python module and keyboard work together to record the the current time of host computer when participants press the keyboard.

#### Data processing

##### Data synchronization

In this study, the raw data of Δ*Hbo* and Δ*HbR*, raw intensity data, participant’s status and driving scenario data were collected, and these data all contain timestamps. The raw intensity data which can be converted into Δ*Hbo* and Δ*HbR* based on the modified Lambert-Beer law. OxySoft receives the raw data of Δ*Hbo* and Δ*HbR* from Matlab/Simulink module with the lab streaming layer platform, then adds the current time of host computer to these data and saves the data; Python module and keyboard work together to record the current time of host computer when they press the keyboard, and the accuracy of current time can reach one-millisecond level. VTD software is deployed in a real-time system which owns outstanding real-time performance. The distance between the host compute and hardware-in-loop equipment is very close and about 5 meters, and they is connected directly by an Ethernet cable, Besides, we tested the time delay by sending data at a frequency of 100 HZ, the maximum, minimum and average values are 8.286, 0.895 and 1.047 milliseconds, respectively. The maximum time delay is smaller than the sampling interval of this human factor signal acquisition system. When Matlab/Simulink module receives driving scenario data using user datagram protocol (UDP), it also adds the current time of host computer to these data. Then the event, vehicle-related, behavioral and fNIRS data can be aligned based on the time of the host computer.

#### Participant

**Fig. 2:**
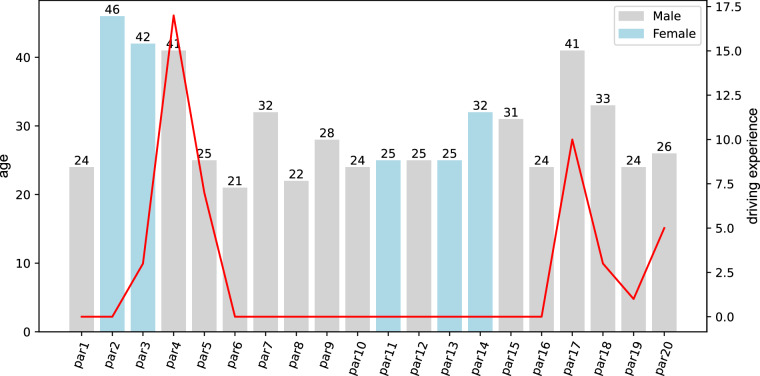
The detailed information about participants.

Twenty participants completed the experiments, and they all are Chinese. Their ages range from 21–46 years, and there are 5 females and 15 males. Participants 3, 4, 5, 17, 18, 19, and 20 have valid driving experience with 3, 17, 7, 10, 3, 1 and 5 years of driving experience, respectively. The ages of ten of the participants are in the range of 20 to 25 years, furthermore, there are 2, 4 and 4 participants whose ages are between 26 to 30 years, between 31 to 35 years, and between 36 to 46 years, respectively. Detailed information on the age, sex and driving experience of the participants are shown in Fig. 2. Bar chart indicates the characteristic of sex and age, blue bar and gray bar indicate female and male, respectively. The height of bar represents age, and red broken line indicates the characteristic of driving experience.

#### Blood oxygen monitoring device

**Fig. 3:**
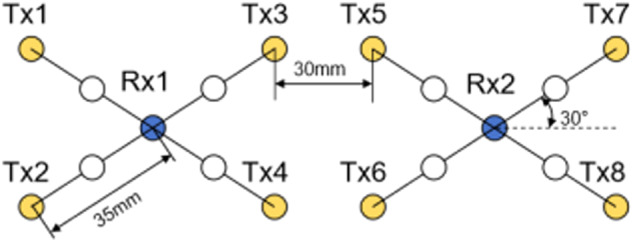
OctaMon Optode Layout.

In those experiments, an OctaMon continuous wave (CW) NIRS device which is manufactured by Artinis, a Dutch company, is used. The device consists of 8 active channels: 8 light emitting diodes serving as transmitters (Tx1-Tx8) with 2 wavelengths (nominal ${\lambda }_{1| 2}=760| 850$ nm), this device is in compliance with the safety standard IEC 62471, and 2 photo diodes as detectors (Rx1 and Rx2) with ambient light protection. The optode position is measured using 3D digitizer system,and Artinis provides a predefined digitization established using 3D digitizer system. With this predefined digitization, the Montreal Neurological Institute (MNI) coordinates of eight channels can be automatically calculated by the OxySoft software (version 3.3.34.1) of Artinis (Table 3). The optode layout is shown in Fig. 3, with an optode distance (d) of 35 mm and a distance of 30 mm between the two middle-top transmitters. The OxySoft software can automatically convert the data into concentration changes based on the modified Lambert-Beer law. The sampling rate of this blood oxygen monitoring device is set to 50 Hz. The sampling rate of this human factor signal acquisition system are 100 Hz, so we made a frequency shift about blood oxygen monitoring device that from 50 Hz to 100 Hz.

**Table 3:** The MNI coordinates of these eight channels.

Channel	Channel 1	Channel 2	Channel 3	Channel 4
MNI coordinates	(57, 50.56, 19.33)	(57.12, 56.1, 1.26)	(38.41, 70.37, 19.56)	(32.83, 76.89, −0.91)
**Channel**	**Channel 5**	**Channel 6**	**Channel 7**	**Channel 8**
MNI coordinates	(−31.45, 75.44, 21.04)	(−33.04, 77.66, 0.59)	(−56.11, 54.37, 19.49)	(−55.73, 60.39, −1)

## Data Records

The dataset^25^ is available at OpenNeuro, and its DOI is 10.18112/openneuro.ds004973.v1.0.1. This dataset contains the raw data which can be converted into Δ*Hbo* and Δ*HbR* based on the modified Lambert-Beer law and split points (stimulus points). the kinetic energy field^26^ is one of the safety indicator^27–29^. The kinetic energy field indicates a danger degree of driving scenario, and it involves relative longitudinal distance and the speed of target vehicle (or pedestrian), and it is shown in (1). In those experiments, the kinetic energy field is adopted as an objective indicator for indicating the danger degree of driving scenario, and the value 0.05 of the kinetic energy field is chosen as the split point (stimulus point) based on experience.

1 $${{\bf{E}}}_{v}=\frac{G{R}_{2}{M}_{2}}{{{\boldsymbol{r}}}^{{k}_{1}}}\frac{{{\boldsymbol{r}}}^{{k}_{1}}}{| {{\boldsymbol{r}}}^{{k}_{1}}| }{e}^{\left[{k}_{2}{v}_{2}cos({\theta }_{2})\right]}$$

where *G*, *k*_1_, *k*_2_, *M*_2_ and *R*_2_ are five constants, *G* = 0.001, *k*_1_ = 1, *k*_2_ = 0.05, *M*_2_ = 1705, and *R*_2_ = 1. The velocity of target vehicle (or pedestrian) is denoted by *v*_2_, ***r*** represents the distance between ego vehicle and target vehicle (or pedestrian), and *θ*_2_ denotes the angle between ***r*** and *v*_2_.

There are three files (participants.tsv, participants.json and dataset_description.json) and twenty folders at this dataset. Those twenty folders respectively save the data from twenty participants in BIDs-complaint format. The file of participants.tsv records age, sex, driving experience and subjective evaluations about dangerous degree of VTD segment. The file of participants.json and dataset_description.json are the explanatory documents of participants and dataset, respectively.

## Technical Validation

**Fig. 4:**
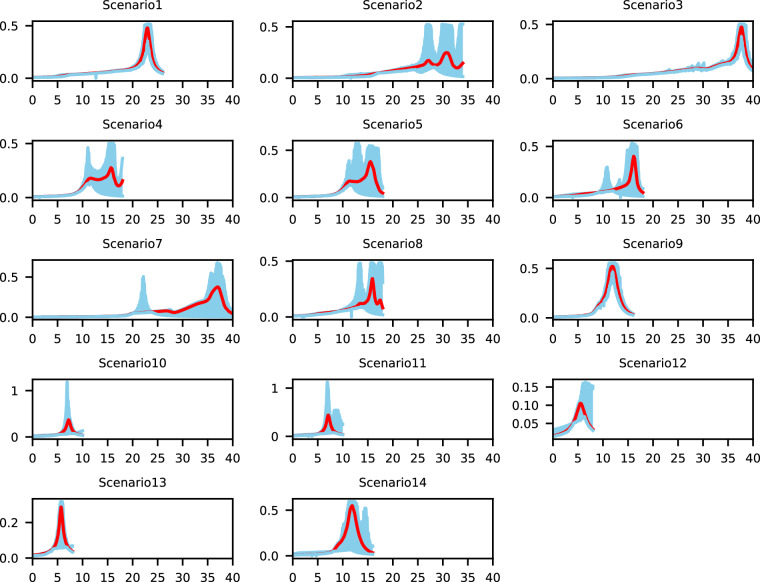
The kinetic energy fields in fourteen types of highly automated driving scenarios.

Those split points also divide one automated driving scenarios into a front-half segment and a later-half segment. The front-half is considered as a low-risk segment, and later-half is considered as a high-risk segment. In these experiments, when all participants complete their tasks, the data from the same highly automated driving scenario is extracted based on Table 1, and regarded as a group. The kinetic energy fields in the fourteen types of highly automated driving scenarios are shown in Fig. 4. The red line in subgraphs represents the mean value of the kinetic energy fields in one scenario, and the sky blue area represents the possible kinetic energy fields in one scenario. Figure 4 shows that there are obvious differences between the kinetic energy fields in low-risk and high-risk segments, and this indicates that the episode division of this dataset is reasonable.

## Usage Notes

This dataset contains the raw intensity data and split data, and is shared at OpenNeuro. Besides, we also provide other tools and information.

- In these experiments, fourteen types of highly automated driving scenarios were built based on VTD software. These twelve VTD segments can be rebuilt using VTD software and a project file. We provide this project file and one video visualizing these driving scenarios.
- The 8-channel fNIRS device used in these experiments can also output the raw Δ*HbO* and Δ*HbR* data, so, we provide four functions in Python to process these raw data of Δ*HbO* and Δ*HbR*. These functions implement the detrending and filtering goals, and the temporal derivative distribution repair^30^ and CBSI algorithms^31^, respectively.
- Homer3^24^ is an open-source software toolkit used for fNIRS, we provide one example of data preprocessing using Homer3 and data analysis using machine learning. The data of one participant in one highly automated driving scenario is stored in a csv file. In this example, the raw Δ*Hbo* and Δ*HbR* data obtained by OxySoft software, the preprocessing Δ*Hbo* and Δ*HbR* data obtained by Homer3, and driving scenario data are available. The driving scenario data consists of the positions, velocities and acceleration of ego vehicle and target vehicle (or pedestrian). Besides, in this example, we also analyses the difference of passengers’ mental activities between low-risk and high-risk segments uisng machine learning algorithms.
- In this study, we provide the MNI coordinates of each data acquisition channel. In order to obtain the location names of each data acquisition channel, we also provide a function which is realized by python. Its name is “aff2tal”, and it can transform MNI coordinates into Talairach coordinates. Then the location names can be obtained using the Talairach coordinate and “Shortcut to TalairachClient” software.
- This dataset provides the data support for analyzing the driving risk cognition of passengers in highly automated driving scenarios. Reinforcement learning (RL) algorithms own learning ability and have been used on highly autonomous vehicles^32–37^. At present, universal decision-making strategies are the focus of related studies, and the distinctive demands of individual passengers are ignored. This dataset provides a foundation for achieving a suitable and unique decision-making strategy for each passenger based on RL algorithms and the mental activities of passengers which are obtained via fNIRS.

## Data Availability

The relational codes and example mentioned in this study and a brief description (readme.md) have been uploaded in github https://github.com/benchidefeng/fNIRS-experiment-for-automated-driving-scenarios.git Or Please contact the corresponding author with any further queries regarding code availability.
